# TLR-adjuvanted nanoparticle vaccines differentially influence the quality and longevity of responses to malaria antigen Pfs25

**DOI:** 10.1172/jci.insight.120692

**Published:** 2018-05-17

**Authors:** Elizabeth A. Thompson, Sebastian Ols, Kazutoyo Miura, Kelly Rausch, David L. Narum, Mats Spångberg, Michal Juraska, Ulrike Wille-Reece, Amy Weiner, Randall F. Howard, Carole A. Long, Patrick E. Duffy, Lloyd Johnston, Conlin P. O’Neil, Karin Loré

**Affiliations:** 1 Department of Medicine Solna, Division of Immunology and Allergy, and; 2 Center for Molecular Medicine, Karolinska Institutet, Stockholm, Sweden.; 3 Laboratory of Malaria and Vector Research and; 4 Laboratory of Malaria Immunology and Vaccinology, National Institute of Allergy and Infectious Diseases, NIH, Rockville, Maryland, USA.; 5 Astrid Fagraeus Laboratory, Karolinska Institutet, Stockholm, Sweden.; 6 Vaccine and Infectious Disease Division, Fred Hutchinson Cancer Research Center, Seattle, Washington, USA.; 7 PATH’s Malaria Vaccine Initiative, Washington, DC, USA.; 8 Bill and Melinda Gates Foundation, Seattle, Washington, USA.; 9 Infectious Disease Research Institute, Seattle, Washington, USA.; 10 Selecta Biosciences Inc., Watertown, Massachusetts, USA.

**Keywords:** Immunology, Vaccines, Adaptive immunity, Innate immunity, Malaria

## Abstract

Transmission-blocking vaccines (TBVs) are considered an integral element of malaria eradication efforts. Despite promising evaluations of *Plasmodium falciparum* Pfs25-based TBVs in mice, clinical trials have failed to induce robust and long-lived Ab titers, in part due to the poorly immunogenic nature of Pfs25. Using nonhuman primates, we demonstrate that multiple aspects of Pfs25 immunity were enhanced by antigen encapsulation in poly(lactic-*co*-glycolic acid)–based [(PLGA)-based] synthetic vaccine particles (SVP[Pfs25]) and potent TLR-based adjuvants. SVP[Pfs25] increased Ab titers, Pfs25-specific plasmablasts, circulating memory B cells, and plasma cells in the bone marrow when benchmarked against the clinically tested multimeric form Pfs25-EPA given with GLA-LSQ. SVP[Pfs25] also induced the first reported Pfs25-specific circulating Th1 and Tfh cells to our knowledge. Multivariate correlative analysis indicated several mechanisms for the improved Ab responses. While Pfs25-specific B cells were responsible for increasing Ab titers, T cell responses stimulated increased Ab avidity. The innate immune activation differentially stimulated by the adjuvants revealed a strong correlation between type I IFN polarization, induced by R848 and CpG, and increased Ab half-life and longevity. Collectively, the data identify ways to improve vaccine-induced immunity to poorly immunogenic proteins, both by the choice of antigen and adjuvant formulation, and highlight underlying immunological mechanisms.

## Abstract 2

Distinct TLR-based adjuvants and nanoparticle protein formulations differentially regulate quality and durability of antibody responses in rhesus macaques.

## Introduction

Nonlive vaccine platforms offer advantages in terms of safety but are often poorly immunogenic without the addition of an adjuvant. A leading trend in vaccine development today is using nanoparticle structures to enhance the immunogenicity of subunit proteins and adjuvants that target the innate immune system via defined TLR agonists. Nanoparticles based on poly(lactic-*co*-glycolic acid) – (PLGA) for example – have been shown to significantly increase humoral and cellular responses (1–4). Mouse models have shown that PLGA nanoparticles can improve targeted antigen trafficking to draining lymph nodes (LNs), increase antigen retention, and improve antigen uptake by DCs and professional antigen-presenting cells (5, 6). These factors benefit antigen presentation to T cells, interaction with B cells, germinal center formation, and, consequently, Ab development (5). In addition, TLR-based adjuvants provide potent means to specifically tune the response to elicit the desired adaptive outcome. Although these technologies have been well established, Ab titers achieved in mouse models do not always translate well into humans. A common problem is the relative lack of mechanistic insights into the development of immune responses from the time of immunization until the elicitation of Ab responses.

Malaria would greatly benefit from optimized protein and adjuvant formulations, as it has so far eluded standard vaccination strategies. While there are several stages during the life cycle of the malaria parasite that can be targeted with vaccination, a promising avenue for malaria elimination and eradication is the development of transmission-blocking vaccines (TBVs). TBVs aim to inhibit parasite development in the mosquito midgut through vaccine-elicited Abs taken up during the blood meal and, therefore, require a high level of antigen-specific Abs that can be maintained over time (7, 8). Of the multiple TBV candidates, *Plasmodium falciparum* protein Pfs25 has advanced the furthest. Pfs25 is expressed on the surface of zygotes during their development into ookinetes, and since this process takes place entirely within the mosquito, this antigen is never expressed in the human host (7). Pfs25 is more highly conserved than other candidates, and a direct correlation between anti-Pfs25 IgG titers and transmission-blocking activity has been established (9), making it an attractive antigen from a vaccine development point of view. In general, monomeric Pfs25 protein has been shown to be poorly immunogenic, but protein formulation and multimerization methods have been able to increase Ab titers (10–13). Although these advancements showed promise in mice, the relatively low Ab titers generated in humans have impeded further progress (14, 15). The most clinically advanced formulation utilized a chemical conjugate, Pfs25-EPA, together with Alhydrogel (InvivoGen) (14). In this clinical trial, induction of transmission-reducing activity required 4 doses, and the activity declined quickly, coinciding with waning Ab titers. Although it is encouraging that vaccination of humans can induce transmission-blocking Abs targeting Pfs25, it is expected that more potent but safe vaccine antigen/adjuvant formulations will be needed to achieve sufficient and durable Ab levels.

In the current study, we aimed to understand the mechanisms underlying vaccination with poorly immunogenic proteins, such as Pfs25, and how to increase the immunogenicity. To investigate this in a physiologically relevant model, we used PLGA-based synthetic vaccine particles (SVPs) codelivered with TLR-based adjuvants in rhesus macaques. To provide a clinical benchmark, SVPs were compared with Pfs25-EPA plus GLA-LSQ. As mentioned above, Pfs25-EPA has already been tested in humans with Alhydrogel (14) and is currently being tested in combination with AS01 (clinical trial ID, NCT02942277). GLA-LSQ is an adjuvant system composed of a liposomal formulation, including QS21 and a synthetic TLR4 agonist, that is, in some respects, similar to AS01. The SVPs were also delivered with GLA-LSQ or coadministered with SVPs containing the TLR ligands CpG or R848 as adjuvants. SVPs containing TLR ligands were previously found to increase safety and immunogenicity by eliciting more localized immune responses compared with free TLR ligands (2). To better understand the development of immunity to the formulations, we analyzed various compartments of the immune response in detail, including innate cell mobilization/activation, innate gene profiling, and the development of B cell and T cell responses. By longitudinally following the animals we found improved immunity with PLGA-based nanoparticle formulation and identified correlates of vaccine immunity, leading to robust, long-lived, functional Ab titers.

## Results

### Study design.

**Figure 1:**
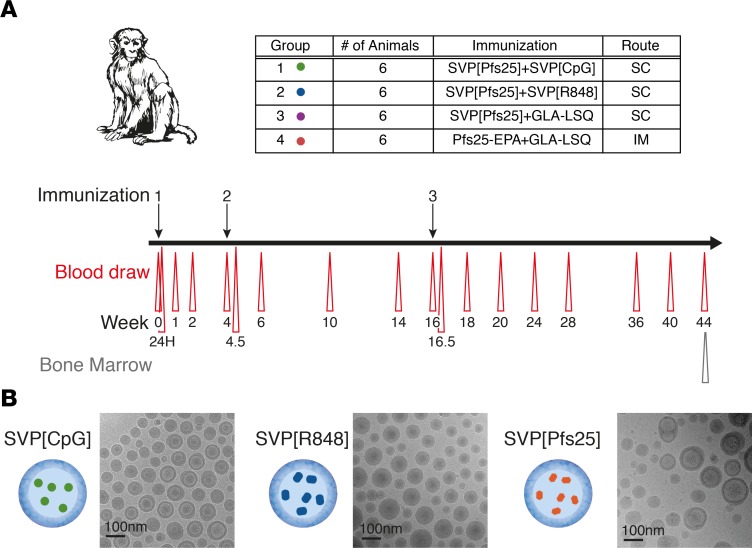
Study design. (**A**) Study design. Four groups of rhesus macaques (*n* = 6/group) were immunized 3 times at week 0, 4, and 16, according to the chart. Blood draws were taken throughout study according to schedule, and bone marrow was collected at study end. (**B**) Transmission electron microscopy images of representative synthetic vaccine particles (SVPs). Scale bar: 100 nm.

Four groups of rhesus macaques (*n* = 6/group) were immunized as shown in Figure 1. The macaques were outbred and a mixture of males and females, reflecting the human population. All groups received the same dose of Pfs25. Groups 1, 2, and 3 received Pfs25 encapsulated in SVPs (SVP[Pfs25]). Groups were adjuvanted with SVPs encapsulating CpG ODN 2395 (SVP[CpG]), resiquimod (SVP[R848]), or GLA-LSQ, respectively. Group 4 received Pfs25-EPA plus GLA-LSQ and served as a clinical benchmark group for the SVP[Pfs25] formulations, as this formulation has shown the most potent responses in mice (16) and is closely related to the Pfs25-EPA plus AS01 formulation currently in clinical trials (NCT02942277). The SVPs were designed to be delivered s.c. to increase trafficking to the LN, whereas the Pfs25-EPA was delivered via the i.m. route, according to historical protocols, to provide translatability to ongoing clinical trials. The animals were immunized with a homologous prime-boost strategy at weeks 0, 4, and 16, and peripheral blood samples were analyzed over the 44-week period.

**Figure 2:**
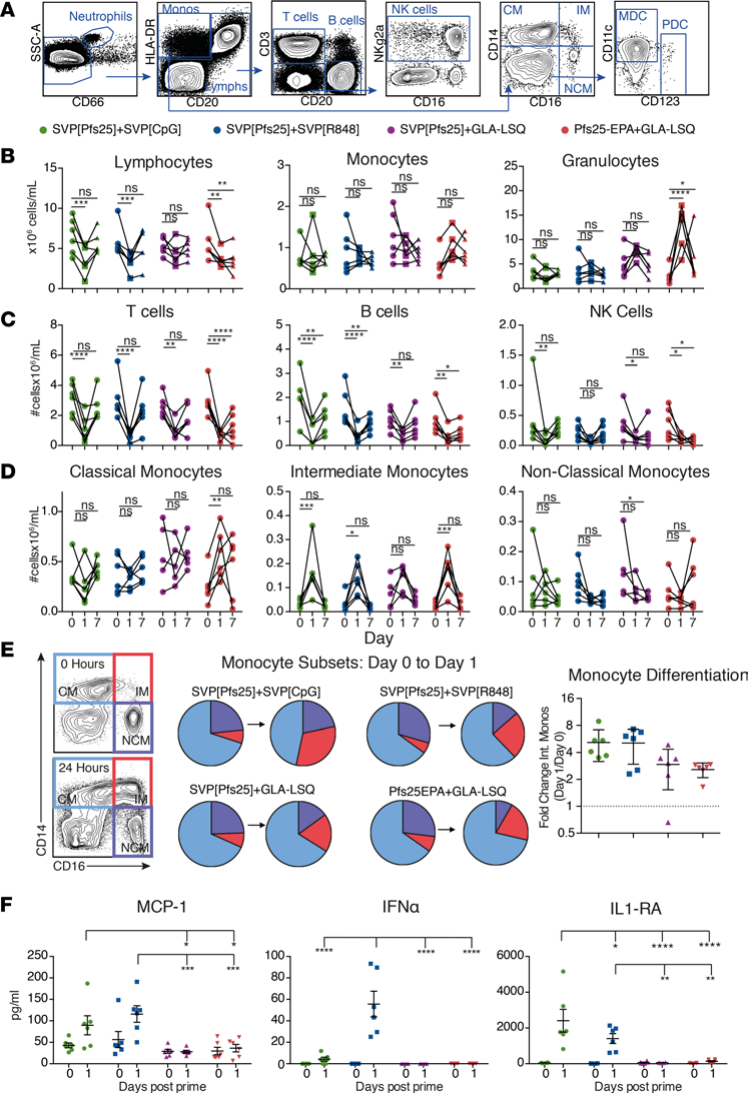
Immunization induces alteration of cellular populations and cytokine production during innate response. (**A**) Representative gating of peripheral blood immune subsets monitored for innate activity by flow cytometry. (**B**) Complete blood counts. (**C** and **D**) Cell subset frequencies normalized to lymphocyte (**C**) or monocyte (**D**) complete blood counts. (**E**) Differentiation of monocyte populations; classical monocytes (CM), intermediate monocyte (IM), or nonclassical monocytes (NCM) are shown as the percentage of total monocytes (pie charts) or fold change of intermediate monocytes from day 0 to 1 (mean ± SEM). (**F**) Plasma cytokines increased on day 1 after immunization (mean ± SEM). Groups were compared using 2-way ANOVA. *P ≤ 0.05; **P ≤ 0.01; ***P ≤ 0.001; and ****P ≤ 0.0001.

*Induction of cell mobilization and IFN-αproduction following immunization*. Since innate immune responses dictate the development of long-term protective responses (17), we performed an extensive analysis of the innate profile induced by the different formulations. All formulations were well tolerated and induced a mild and transient elevation in liver enzymes, similar to known vaccinations (18, 19), but no increase in body temperature (Supplemental Figure 1; supplemental material available online with this article; https://doi.org/10.1172/jci.insight.120692DS1). By flow cytometry (Figure 2A) and complete blood count analysis (Figure 2B), we found that the number of lymphocytes rapidly and transiently declined in the circulation in all groups 24 hours after immunization (Figure 2C), likely reflecting immune activation and migration to lymphoid tissue (20). There were limited changes in the numbers of DC subsets (Supplemental Figure 2), but there was a marked increase of intermediate CD14^+^CD16^+^ monocytes, both in absolute numbers and as a proportion within the monocyte compartment (Figure 2, D and E). This indicates a differentiation of classical CD14^+^ monocytes into intermediate monocytes, as described in response to vaccine adjuvants containing TLR ligands (20, 21). The CpG- and R848-adjuvanted groups showed the most dramatic level of monocyte differentiation, whereas GLA-LSQ–immunized animals showed a trend toward higher monocyte numbers in general (Figure 2E). Phenotypic maturation of DCs or monocytes involving upregulation of costimulatory markers was not detected in the blood at this time (Supplemental Figure 2B), although differentiation of intermediate monocytes reflects myeloid cell activation. Additionally, systemic activation was measured using the Cytokine & Chemokine 30-Plex NHP ProcartaPlex Panel (eBioscience). The majority of analytes measured showed no alteration from baseline, consistent with previous findings, where SVPs encapsulating R848 induced cytokines locally in the vaccine-draining LN but not systemically (2). Animals receiving SVP[CpG] or SVP[R848], but not GLA-LSQ, showed increased levels of MCP-1, IFN-α, and IL1-RA (Figure 2F). We and others have previously shown that TLR7/8 and TLR9 agonists, but not TLR4 agonists, induce type I IFN responses (20, 22, 23). Collectively, this demonstrates that all formulations induced strong innate immune activation characterized by lymphoid cell mobilization and the groups receiving CpG and R848 showed a higher magnitude of systemic innate activation, such as monocyte differentiation and cytokine production.

### Adjuvant-driven changes in gene expression after immunization.

**Figure 3:**
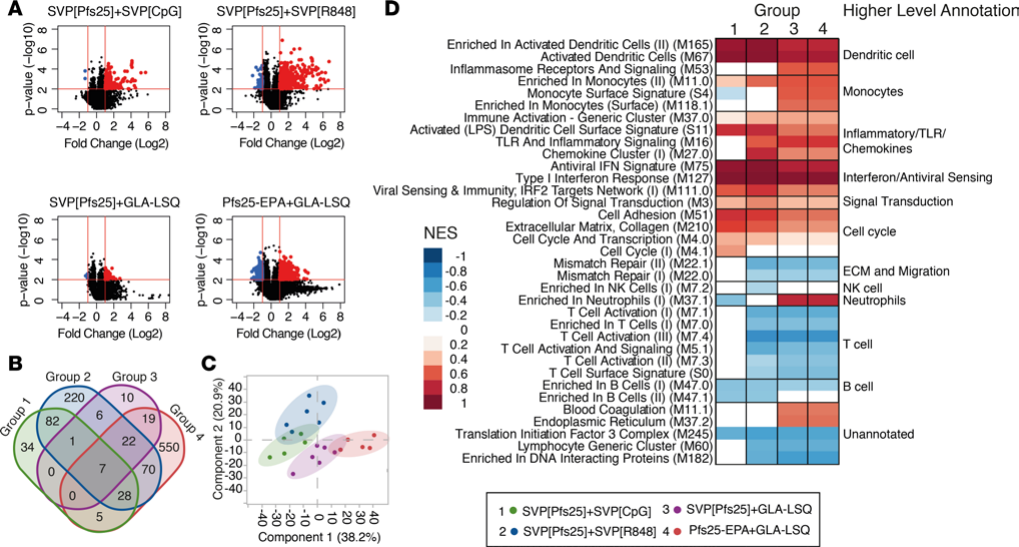
Modulation of gene expression following immunization. (**A**) Differentially expressed genes displayed as volcano plots. Criteria used are raw *P* < 0.01, calculated using a paired 2-tailed Student’s *t* test, and fold change (FC) > 2 from day 0 to day 1. (**B** and **C**) Genes showing *P* < 0.01 and FC > 2 in any of the groups were selected for further analysis (1,103 genes). (**B**) Venn diagram depicting the number of overlapping genes between each group for selected genes. (**C**) Principal component analysis using individual FC values for selected genes. (**D**) Each box represents a blood transcript module (BTM) and colors represent the normalized enrichment score (NES) after gene set enrichment analysis using all genes. BTMs are grouped into higher level annotation as indicated on the right.

In line with the noted innate activation, transcriptomic analyses of blood collected 24 hours after immunization showed significant alteration of gene expression compared with matched prevaccination samples in all groups (Figure 3A; accession GSE10290). Since all animals were naive to Pfs25, transcriptomic changes at this early time point should be driven by innate stimulation provided by adjuvants and not protein formulation. Animals receiving Pfs25-EPA plus GLA-LSQ showed the highest number of modulated genes, likely reflecting differences in kinetics associated with the route of administration, since GLA-LSQ delivered s.c. induced a lower number of differentially expressed genes than i.m. delivery. However, the difference in gene expression (fold change) was on average higher after immunization with R848, despite having a lower number of significantly altered genes. Genes that were highly altered (*P* < 0.01 and fold change >2; 1,103 genes) were selected to further characterize the transcriptomic profiles induced by each formulation (Supplemental Figure 3A). There were only 7 genes commonly altered by all groups; however, there was considerable overlap between the CpG and R848 groups (Figure 3B). In contrast, a majority of the genes (550 of 701) that were differentially expressed in the Pfs25-EPA plus GLA-LSQ group were unique to this group (Figure 3B). Furthering this notion, PCA analysis (Figure 3C) and hierarchical clustering (Supplemental Figure 3B) revealed a distinction in the innate profiles driven by SVP[R848] and SVP[CpG] compared with those of the GLA-LSQ–immunized animals. To compare functional pathways altered by vaccination within the different groups, we used gene set enrichment analysis using blood transcription modules as gene sets (24). Within each vaccination group, genes were ranked by the average fold change from baseline and then evaluated for functional pathways overrepresented or underrepresented after vaccination (Figure 3D). Modules associated with innate activity, including DC activation and inflammatory/TLR signaling, were in general highly upregulated. The downregulation of monocyte surface signature (S4) in the SVP[R848] group may reflect a differentiation away from the classical monocyte surface phenotype. Interestingly, there was an enrichment of antiviral IFN signaling with all adjuvants (Figure 3D), although detectable IFN-α was only seen in the periphery of animals receiving R848 or CpG, and this was reflected in the higher fold change differences in IFN-related genes in these groups (Supplemental Figure 4D). Further, modules associated with specific cell subsets coincided with the altered cell frequencies in the circulation (Figure 2, B and C). Neutrophil modules were increased only in the GLA-LSQ groups, and there was a decrease in lymphocyte-associated modules across all groups.

### Robust and sustained Ab titers with SVP immunization.

**Figure 4:**
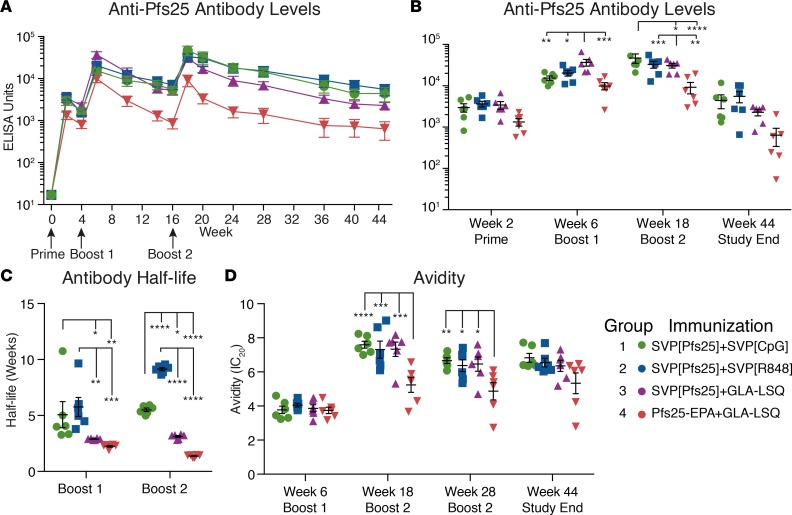
Robust and sustained Ab titers. (**A**) Ab titers against Pfs25 were measured using a standardized ELISA. (**B**) Ab titers at peak immunogenicity after immunizations and study end. (**C**) Ab titer half-life was calculated following boost 1 (week 6 to week 16) and boost 2 (week 18 to week 44). (**D**) Ab avidity was calculated using a modified urea ELISA. Urea concentration that dissociated 20% of bound anti-Pfs25 IgG is shown (IC_20_ values). All data are represented as mean ± SEM. Groups were compared using 2-way ANOVA. *P ≤ 0.05; **P ≤ 0.01; ***P ≤ 0.001; and ****P ≤ 0.0001.

Immunization with all tested formulations induced detectable Pfs25-specific IgG after the prime immunization, and titers markedly increased with boost immunizations (Figure 4A). The group receiving SVP[Pfs25] plus GLA-LSQ showed the highest peak Ab titers after the first boost immunization, although animals receiving CpG or R848 showed comparable peak responses after the second boost (Figure 4B). Overall, the Pfs25-EPA group showed the lowest titers. A common problem with nonlive vaccine platforms is the difficulty maintaining high Ab titers over time. Ab decay typically follows a biphasic pattern, in which the first period of decay reflects the half-life of the Abs (around 20 days) and the robustness of the plasmablast response, while the second phase is dependent on long-lived plasma cells (LLPCs) in the bone marrow (25, 26). Consistent with this pattern, we saw faster decay rates directly after immunization until a relative plateau effect was achieved approximately 10 weeks later. We therefore used a biphasic model to calculate the half-life of anti-Pfs25 IgG following immunization with the different formulations (Figure 4C). Although the SVP[Pfs25] plus GLA-LSQ group initially showed the highest titers, the animals receiving CpG- or R848-adjuvanted SVP[Pfs25] showed a significantly longer half-life after boost 1 and 2 (Figure 4C). Over the 6-month period following boost 2, animals receiving SVP[R848] had an Ab half-life of approximately 9 weeks, which was 6 times higher than that of the Pfs25-EPA plus GLA-LSQ group and significantly higher than all groups. Since both groups receiving GLA-LSQ showed faster decay, the innate profile driven by the different adjuvants likely affects the induction and longevity of B cell responses. Ab avidity, measured by a urea-dissociation ELISA (Figure 4D), showed that there was no difference between the groups after the first boost. The avidity increased significantly after the second boost, and the SVP formulations showed higher avidity than Pfs25-EPA. Together, the SVP[Pfs25] formulation induced higher titers and avidity than Pfs25-EPA, and the CpG and R848 adjuvants induced a longer Ab half-life than GLA-LSQ.

### Induction of plasmablasts with distinct phenotypes depending on immunization.

**Figure 5:**
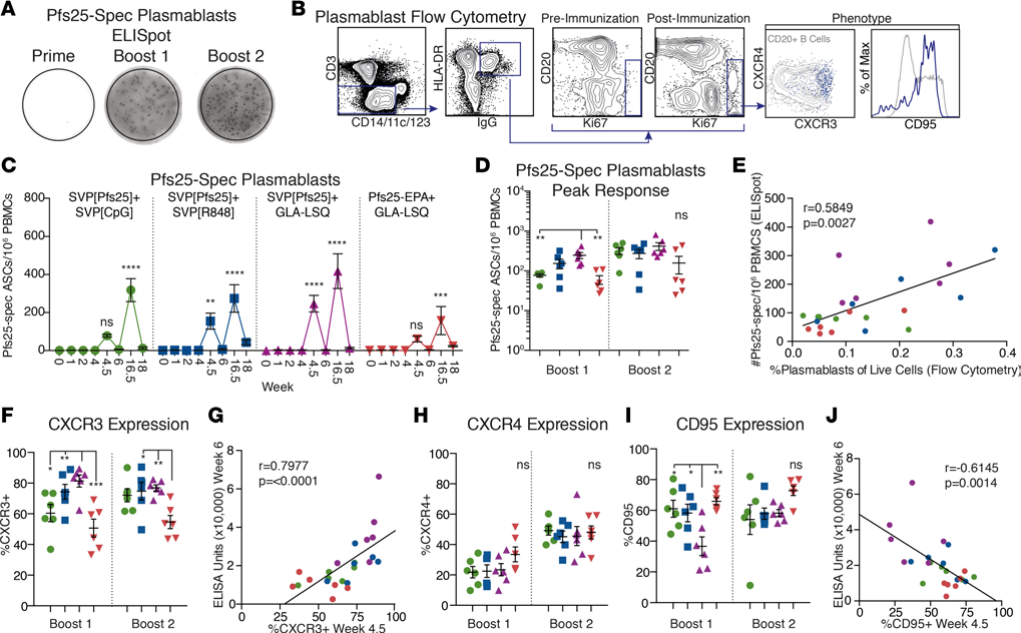
Induction of plasmablasts following boost immunizations with distinct phenotypes. (**A**) Representative examples of ELISpot results from wells coated with Pfs25 protein for enumeration of Pfs25-specific Ab-secreting cells (ASCs; plasmablasts). (**B**) Gating scheme to evaluate kinetics and phenotype of plasmablasts over time using flow cytometry. (**C**) The magnitude of Pfs25-specific plasmablasts evaluated by ELISpot over time. (**D**) Summary of peak responses, 5 days after boost 1 (week 4.5) and 4 days after boost 2 (week 16.5), evaluated by ELISpot. (**E**) Correlation of plasmablasts, as determined by ELISpot (*y* axis) and flow cytometry (*x* axis). (**F–J**) Phenotype of plasmablasts determined by flow cytometry. (**F**) Percentage of plasmablasts expressing CXCR3. (**G**) Correlation of the percentage of CXCR3^+^ plasmablasts at week 4.5 with Ab titers at week 6. (**H**) Percentage of plasmablasts expressing CXCR4. (**I**) Percentage of plasmablasts expressing CD95. (**J**) Correlation of the percentage of CD95^+^ plasmablasts at week 4.5 with Ab titers at week 6. All data represent mean ± SEM, unless otherwise noted. Groups were compared using 2-way ANOVA. Correlation analysis performed using nonparametric Spearman’s test with 2-tailed *P* value. *P ≤ 0.05; **P ≤ 0.01; ***P ≤ 0.001; and ****P ≤ 0.0001.

To better understand the development and maintenance of Ab titers, we evaluated the dynamics of multiple Pfs25-specific B cell subsets. Plasmablasts that transiently appear after immunization were first evaluated by ELISpot for antigen specificity (Figure 5A) and stained directly for phenotyping by flow cytometry (Figure 5B). As expected, prime immunization did not generate detectable Pfs25-specific plasmablasts (27), but they could differentiate from memory B cells during boost immunizations and were readily detectable 4–5 days after boost 1 and 2 (Figure 5C). After the first boost, animals immunized with SVP[Pfs25] plus GLA-LSQ showed the highest numbers of plasmablasts, in line with the highest plasma Ab titers found in this group at the time (Figure 5D). Following the second boost, the groups equalized, although the majority of the Pfs25-EPA–immunized animals showed lower responses. These results, together with the Ab titers, suggest that GLA-LSQ is the stronger adjuvant for priming naive or low-level responses. In contrast, CpG and R848 adjuvants may be more potent at expanding an already existing vaccine-specific memory pool, as seen after boost 2. Such improved boosting capacity may be due to their ability to directly target TLR7 and TLR9 on memory B cells (28). Unlike in mice, human and rhesus B cells have no or low functional expression of TLR4 and would, therefore, have limited responsiveness to direct stimulation by GLA (29).

Enumeration of plasmablasts by flow cytometry closely mirrored the results of Pfs25-specific plasmablasts determined by ELISpot (Figure 5E). Plasmablasts were phenotyped for multiple markers, including their expression of the general tissue-homing marker CXCR3, bone marrow–homing marker CXCR4, and proapoptotic marker CD95 (Figure 5B). While not definitive, these markers indicate the fate of the cells induced by the vaccine (30). We found that a large proportion of plasmablasts expressed CXCR3, in particular in the SVP[Pfs25] groups (Figure 5F). Controlling for the adjuvant effect using GLA-LSQ, there was significantly higher CXCR3 expression in the group immunized with SVP[Pfs25] compared withPfs25-EPA (Figure 5F). However, since the SVPs were administered s.c. and Pfs25-EPA i.m., the difference may be a result of either protein formulation or route of administration. Nevertheless, there was a strong correlation between the percentage of CXCR3^+^ plasmablasts and the Ab titer (Figure 5G). In all groups, the expression of CXCR4 increased from the first boost to the second boost, but there were no significant differences between groups (Figure 5H). CD95 showed distinct patterns depending on the immunization group (Figure 5I), and CD95 expression negatively correlated with Ab titers (Figure 5J). Interestingly, the Pfs25-EPA group with the lowest plasmablast numbers and Ab titers showed the highest expression of CD95, suggesting that the plasmablasts in this group were more prone to cell death and, therefore, may produce Abs over a shorter period than the SVP groups.

### Functional transmission-blocking Ab titers reflect memory B cell and LLPC pool.

**Figure 6:**
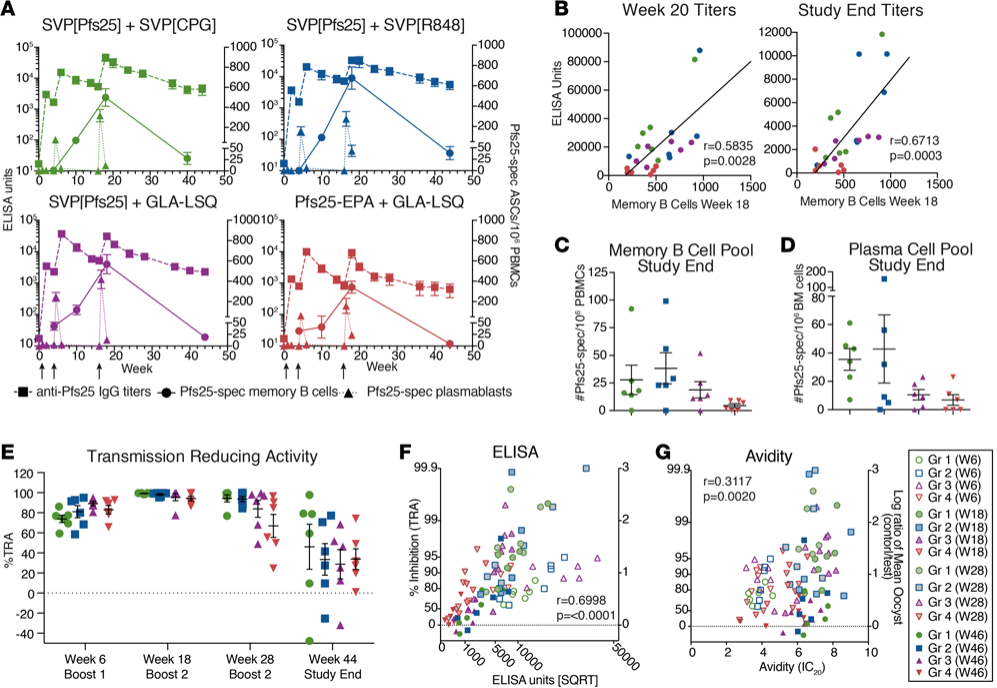
Maintenance of B cell memory and Ab functionality. (**A**) Overview of B cell kinetics throughout the study, including induction of Ab titers (left, *x* axis) and Pfs25-specific plasmablasts and memory B cells (right, *y* axis) determined by ELISA or ELISpot. (**B**) Correlation of memory B cells determined by ELISpot at week 18 with Ab titers at week 20 and study end. (**C**) Peripheral memory B cell pool at study end determined by ELISpot. (**D**) Bone marrow (BM) plasma cell pool at study end determined by ELISpot. (**E**) Ab functionality, as determined by standard membrane feeding assay (SMFA). Shown is the percentage inhibition of oocyst development in the mosquito midgut. TRA, transmission reducing activity. (**F**) Ab titer (ELISA Units, square root) correlated with transmission-reducing activity. (**G**) Ab avidity (inhibitory concentration 20% [IC_20_]) values correlated with transmission-reducing activity. All data represent mean ± SEM, unless otherwise noted. Groups were compared using 2-way ANOVA. Correlation analysis performed using nonparametric Spearman’s test with 2-tailed *P* value.

Plasmablasts, which appear transiently after vaccination, can produce large amounts of Ab, increasing the peak Ab titers. On the other hand, memory B cells and LLPCs are critical for the long-term maintenance of Ab titers. As described above, plasmablast numbers peaked transiently following each boost immunization, preceding increases in Ab titers (Figure 6A). In contrast, we found that the Pfs25-specific memory B cell pool expanded following each immunization and correlated with the ensuing Ab titer (Figure 6, A and B). Consequently, the higher Ab titers found in the SVP[Pfs25] groups were linked to a larger memory B cell pool at study end (Figure 6C). This would likely have direct implications for the feasibility of boosting the responses at a later time to maintain high Ab titers and transmission-blocking efficacy. A proportion of antigen-specific B cells take up residency in the bone marrow as LLPCs, providing the longevity of Ab titers. Since Pfs25 cannot rely on natural boosting to increase Ab titers after exposure, LLPCs will be essential to maintain titers over time. When evaluating the bone marrow compartment at study end, we found that all groups had animals with detectable Pfs25-specific plasma cells, but animals in the groups receiving CpG or R848 adjuvants showed, on average, higher levels of these plasma cells than the GLA-LSQ–adjuvanted groups, mirroring the increased Ab half-life elicited with CpG or R848 adjuvants (Figure 6D).

Given the high level of sustained Ab titers, the functional capacity of the Abs to block parasite development in mosquitos was measured using the standard membrane feeding assay (SMFA) (9). This method has been widely utilized to estimate the transmission-blocking potential of vaccine-induced Abs in preclinical and clinical studies and provides a unique capacity to compare the efficacy of different vaccine platforms. In the SMFA, IgG purified from plasma before or after immunization is mixed with *P*. *falciparum* gametocytes and fed to *Anopheles* mosquitos. The neutralization potential of the Abs can then be quantified by enumerating oocysts’ development in the mosquito midgut (9). All groups showed functional Abs with detectable transmission-reducing activity after the first boost immunization, and, following the second boost, almost all animals showed close to 100% inhibition regardless of group (Figure 6E). Due to the high levels of inhibition after the second boost, the SMFA was titrated to a lower IgG concentration to tease out the differences in functionality between the groups, and these values were used for the subsequent correlations (Supplemental Figure 5A). Although there were no significant differences, there was a trend toward higher inhibition with SVP[Pfs25]. Animals receiving SVP[CpG] or SVP[R848] maintained higher levels of SMFA activity through week 28, and these groups showed lower levels of variation in activity. However, by study end, the titers had dropped to levels where there were no significant differences in SMFA activity between the groups. We found that not only did Ab titers show a strong correlation with SMFA activity as expected (Figure 6F), but avidity also correlated with SMFA activity (Figure 6G). Using a multiple linear regression model, Ab titer, avidity, and immunization group all contributed significantly to explain SMFA activity (Supplemental Figure 5B).

### SVP formulation increased Pfs25-specific T cell help and germinal center activity.

**Figure 7:**
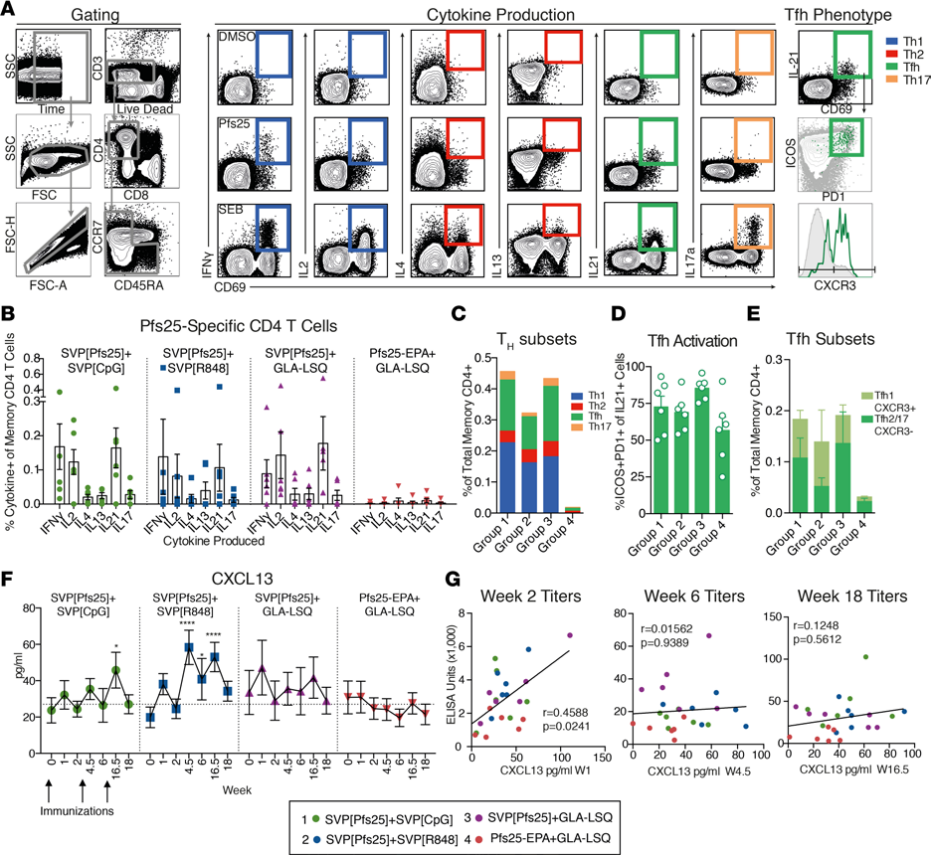
Induction of Pfs25-specific CD4 T cells and germinal center activation. (**A**) Representative gating (live/CD3^+^, CD4^+^/CD8^–^, CD45RA^+^, and CCR7^+^ naive cells gated out), cytokine production, and peripheral Tfh phenotype following overnight stimulation with DMSO, Pfs25 overlapping peptides (15-mers overlapping by 11 peptides**)**), or staphylococcal enterotoxin B in the presence of brefeldin A. (**B**) Quantification of CD4 memory T cells following boost 2 (week 18) producing IFN-γ, IL-2, IL-4, IL-13, IL-21, or IL-17 following Pfs25 peptide stimulation, with negative control (DMSO) values subtracted. (**C**) Induction of Th subsets based on cytokine production; see representative gating. (**D**) Percentage of peripheral Tfh cells with activated phenotype that are ICOS^+^, and PD1^+^. (**E**) Tfh subsets based on CXCR3 expression. (**F**) CXCL13 was measured in plasma following immunization as a biomarker for germinal center activity. Significance was calculated using 2-way ANOVA compared with baseline. (**G**) Correlation of CXCL13 levels with anti-Pfs25 titers. All data represent mean ± SEM, unless otherwise noted. Correlation analysis performed using nonparametric Spearman’s test with 2-tailed *P* value. *P ≤ 0.05 and ****P ≤ 0.0001.

Following immunization, induction of antigen-specific Th cells and strong germinal center (GC) responses in the vaccine-draining LN is a critical event, where B cells interact with cognate T cells and undergo affinity maturation to induce potent Ab responses (31). In this study, we refrained from removing LNs to avoid any risk of interfering with the vaccine response and instead used other measures for induction of T cell responses and GC activity. We used an intracellular cytokine recall assay to evaluate the induction of peripheral Pfs25-specific CD4 T cells and their ability to produce IFN-γ, IL-2, IL-4, IL-13, IL-21, or IL-17a (Figure 7A). Two weeks after the final boost, a majority of animals in the SVP-immunized groups showed detectable Pfs25-specific CD4 T cell responses, in contrast to the Pfs25-EPA–immunized animals, which overall showed low responses (Figure 7B). Some animals, especially in the Pfs25-EPA group, did not induce detectable T cell responses. This is similar to the lack of T cell responses observed in C57BL/6 mice, which were unable to bind Pfs25 via the I-A^b^ MHC class II molecule (16). However, in contrast to C57BL/6 mice, rhesus macaques are genetically outbred, and SVP[Pfs25] induced high Ab titers in all animals in our study that received it, without the need for carrier proteins.

The responding T cells were predominantly Th type I (Th1) cells producing IFN-γ/IL-2 or circulating T follicular helper (Thf) cells producing IL-21 CD4 T cells (Figure 7, B and C). Tfh cells are specialized in providing B cell help and critical for the GC reaction. Tfh cells in LNs have counterparts in the circulation that can be defined by the expression of CXCR5 and a combination of other surface markers (32) or their IL-21 production (33). Circulating Tfh cells are expanded during a brief window, approximately 1 week after vaccination, and during this time can be identified by their surface phenotype. However, ex vivo stimulation combined with staining for IL-21 allows for identification of antigen-specific circulating CD4 T cells transcriptionally similar to Tfh cells found in LNs (33). Circulating Tfh cells can be further subdivided based on their expression of CXCR3 into either Tfh1 (CXCR3^+^) or Tfh2/Tfh17 (CXCR3^–^) cells, both associated with a supportive function of B cell responses and inducible after vaccination (33–36). We found that all formulations induced circulating Tfh cells that primarily expressed the activated phenotype of ICOS^+^PD1^+^ and generated a mixture of Tfh1 and Tfh2/Tfh17 cells (Figure 7, D and E), indicating engagement of GC and Tfh cell help.

CXCL13, the chemokine ligand for CXCR5, is central in structuring GC development and has recently been described as a plasma biomarker for GC activity (37). We found that the levels of CXCL13 in plasma over the course of immunization mimicked the induction of plasmablasts, i.e., transient peaks 1 week after each immunization (Figure 7F). Animals immunized with the SVP[Pfs25] formulations showed higher overall levels of CXCL13 than animals immunized with the Pfs25-EPA formulation, and the adjuvant SVP[R848] showed the highest levels. CXCL13 levels correlated with Ab titers after prime but not boost (Figure 7G). This discrepancy could reflect that GC formation is critical for dictating Ab production following the first antigen encounter; however, following boost immunizations, there is a greater contribution from preexisting memory pools and LLPCs (38). Together, the induction of CXCL13 and circulating Tfh cells indicates increased GC activity in the SVP groups.

### Cluster analysis reveals parameters significantly correlating with Ab titer, longevity, and avidity.

**Figure 8:**
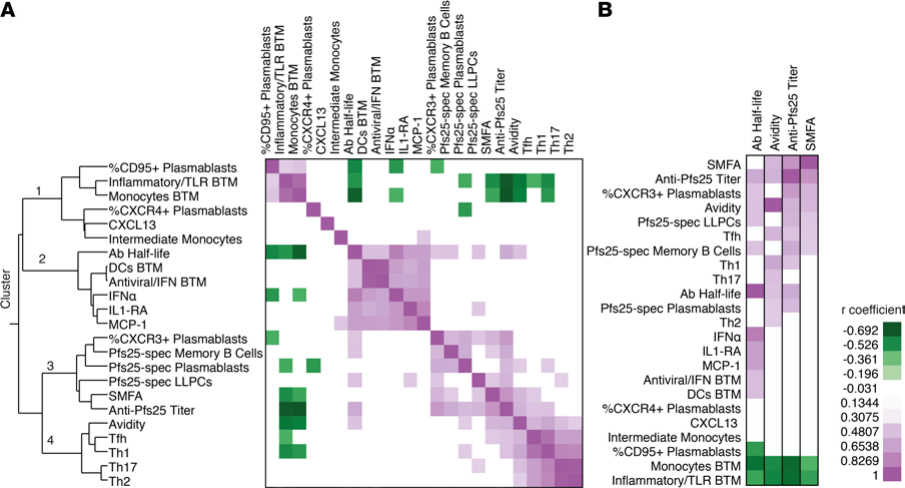
Clustering of significantly correlated values. (**A**) Twenty-three parameters measured in the study at peak immunogenicity (Supplemental Table 1) were analyzed using a multivariate nonparametric Spearman’s test and 2-tailed *P* value. Correlations with a nonsignificant *P* value (>0.05) had Spearman’s coefficient changed to 0, and remaining values underwent 2-way hierarchical clustering. Heatmap showing correlation coefficient with clusters denoted. (**B**) Isolation of correlation values of indicated parameters that correlate with Ab half-life, avidity, titer, and standard membrane feeding assay (SMFA) activity.

Collecting multiple longitudinal samples and performing a variety of immunological assays throughout the study gave us a unique opportunity to better understand what factors influence the induction of long-lived Ab titers and transmission-blocking activity. We therefore performed multivariate nonparametric correlations using all 23 variables measured in this study for each parameter (Supplemental Table 1), followed by hierarchical clustering as described previously (39) (Figure 8A). Since SMFA activity reached an upper threshold at week 18, the data generated using a lower concentration of IgG was used to better differentiate responses of the animals. This analysis revealed 4 distinct clusters. Of note, cluster 3 showed high correlations between Ab titer and SMFA as expected. Additionally, cluster 3 included memory B cells, plasmablasts (in particular, CXCR3^+^ plasmablasts), and LLPCs, demonstrating the close relationship between induction of B cell responses and SMFA activity. Cluster 2 showed a strong correlation of innate cytokine production and IFN gene modulation with Ab half-life, indicating that, while formulation in SVPs may play a strong role in increasing titers, innate activation due to adjuvants may play a larger role in fine-tuning the longevity of the response. Cluster 4 primarily demonstrated that CD4 T cell responses correlate with themselves (i.e., animals that generated large Th1 responses also generated large Tfh responses) but also the strong connection between the induction of T cell responses and Ab avidity, possibly reflecting increased GC engagement and higher quality Ab responses. The primary endpoints of this study (Ab titer, Ab half-life, Ab avidity, and SMFA) were also isolated to better visualize variables that showed significant correlations (Figure 8B). In summary, increased B cell responses were highly correlated with increased titers; innate cytokine production predicted the longevity of the Ab response; and induction of antigen-specific T cell responses (primarily Th1 and Tfh) indicated increased Ab avidity. Together, these measurements provide a point of reference for a variety of nonlive vaccine platforms.

## Discussion

Human clinical trials have demonstrated the feasibility of inducing transmission-blocking Abs; however, their limited success highlights the importance of testing novel protein formulations and stronger adjuvants in a model more clinically relevant than mice. It is important to characterize in detail how both vaccine antigen composition and adjuvants lead to induction of high and durable titers. Therefore, we performed a comparative study that, for the first time to our knowledge, characterized the immune responses leading to transmission-reducing activity in an nonhuman primate (NHP) model using the most clinically advanced vaccine candidate, Pfs25-EPA, and applied a two-pronged approach to further improve immunogenicity. We found that administration of Pfs25-EPA adjuvanted with GLA-LSQ showed a marked increase in responses over previously reported formulations in Alhydrogel in humans and was able to induce strong transmission-reducing activity (14). However, Pfs25 formulated in PLGA SVPs generated higher Pfs25-specific CD4 T cell, plasmablast, and memory B cell responses and, ultimately, higher Ab titers, avidity, and SMFA activity. The adjuvants SVP[R848] and SVP[CpG] were able to further augment the longevity of responses through increased Ab half-life and LLPC maintenance in the bone marrow, with SVP[R848] showing the most dramatic increase. The greater longevity correlated with innate activation, characterized by IFN gene signaling and cytokine production. The correlations established here among a variety of immune responses following immunization with multiple TLR-based adjuvants and protein formulations provide mechanistic insights into improving nonlive vaccination strategies and indicate several ways immunogenicity to Pfs25 can be increased for clinical implementation.

Although subunit protein vaccines offer exceptional safety profiles, they often fail to induce robust and durable immunity, as was seen with Pfs25-EPA and Alhydrogel (14). We found that packaging Pfs25 in a PLGA SVP augmented several aspects of the immune response when compared with Pfs25-EPA. Although Pfs25-EPA forms nanoparticle-like structures comparable in size to the RTS,S virus-like particle, they are still relatively heterogeneous (12). There is a well-documented correlation between antigen size and transport via lymphatics (5). Therefore, the size of SVPs (approximately 130 nm) allows antigen to efficiently target draining LNs after s.c. administration (6). The slowly degrading PLGA nanoparticles can then increase antigen retention within the LN. Indicative of improved antigen trafficking and presentation in the LN, we observed higher GC activity in animals receiving the SVP formulations, as assessed by CXCL13, induction of Tfh cells, and increased Ab avidity. Nanoparticles can also increase B cell responses by displaying antigen in a predetermined array, best suited for BCR cross linking (40). This is unlikely to be a major reason behind the increased Ab titers in the present study, as Pfs25 was loaded into the SVPs but was not conjugated to the surface in a fixed manner. However, it opens up the intriguing possibility that Ab responses could be further augmented using SVP technology with antigen arrayed on the surface. Instead, increased immunogenicity was likely driven by a combination of increased antigen trafficking to and retention in LNs and antigen uptake by DCs. The enhanced potential for antigen uptake and presentation to T cells using the SVP formulation was demonstrated by the ability to induce substantial Pfs25-specific CD4 T cells only in the SVP formulations.

CD4 T cell engagement is essential for long-lived isotype-switched Ab responses. The high levels of anti-Pfs25 IgG suggest that CD4 T cell help was provided in all groups. Although we detected weak Pfs25-specific T cell responses with Pfs25-EPA, there were likely responses against the carrier protein that are similar to those reported in C57BL/6 mice (16). Further, while the poor immunogenicity of Pfs25 is illuminated by the lack of MHC-binding epitopes in C57BL/6 mice, this prediction overlooks the highly diverse MHC expression in humans and NHPs, which has been shown to have a large effect on vaccine-elicited T cell responses in different mouse strains (29). Some animals in our study were not able to generate detectable T cell responses to Pfs25, potentially indicating a poor MHC haplotype for presentation of Pfs25 epitopes. Since both Pfs25-specific Th1 and Tfh cells correlated with increased titers and clustered with Ab avidity, it will be important to design vaccine platforms that can generate robust T cell responses across a variety of MHC haplotypes. The Pfs25-EPA formulation is able to overcome the low immunogenicity by engaging CD4 T cell help via a carrier protein; however, we demonstrate here that when using an SVP formulation, a carrier protein is not essential.

The magnitude and phenotype of B cell responses after immunization with Pfs25-EPA and SVP formulations differed. In general, B cell responses were higher after immunization with SVP[Pfs25], but differences between groups were limited at peak immunogenicity. However, the two formulations induced distinct phenotypes of circulating plasmablasts. SVP[Pfs25] induced plasmablasts with high CXCR3 expression and low CD95 expression. This phenotype correlated with increased Ab titers, potentially reflecting an improved survival potential after homing to tissues via CXCR3, and low expression of the proapoptotic marker CD95. In the present study, it was not possible to determine whether the distinct phenotypes are a result of protein formulation or route of delivery. However, the route of delivery should be included as a parameter of vaccine formulation that can alter vaccine responses.

Using 3 distinct TLR-based adjuvants, we could dissect the ways adjuvants differentially regulate immune responses. Due to the differences in mouse versus human/NHP TLR expression (41), it is important to perform this type of analysis in NHPs. There was a clear induction of robust Ab titers and transmission-reducing activity in animals receiving Pfs25-EPA plus GLA-LSQ, confirming that targeting TLR4 would be an effective route for inducing transmission-reducing activity in NHPs, as seen previously in mice (16). However, we found that GLA-LSQ was better at priming Ab responses, while CpG and R848 were superior in boost immunizations. It would therefore be interesting in future studies to evaluate whether the titers would further increase by using GLA-LSQ as the prime and CpG or R848 in the boost immunizations. We hypothesize that this difference is due to a combination of the ability to (a) directly target memory B cells via TLR7/8 and TLR9 in NHPs and (b) induce a stronger innate profile, primarily characterized by IFN gene signaling and IFN-α production. Although we did not find detectable IFN-α production with GLA-LSQ, both AS01 and QS-21 formulated in liposomes have been shown to modulate IFN-related genes in humans and mice, respectively (42, 43), and AS01 induced IFN-related chemokines, such as CXCL10, in the draining LNs and injection sites of mice (44). Nevertheless, genes associated with antiviral signaling showed the highest fold change in the SVP[R848] group, reflecting the highest levels of IFN-α in circulation in this study.

IFN-α showed a strong correlation with increased Ab half-life, most clearly demonstrated with SVP[R848]. In comparison, the half-life induced by SVP[R848] was longer than those observed following immunization with HIV-Env formulated with 8 distinct adjuvants in rhesus macaques, using the same biphasic model for calculation (45). GLA-LSQ, which demonstrated a relatively low Ab half-life, showed approximately the same half-life as unadjuvanted Env or Env with alum. In line with our data, the TLR3 agonist poly IC:LC induced the longest half-life and showed the most robust induction of IFN-α. We have previously shown that IFN-α produced after TLR7/8 or TLR9 stimulation can modulate B cell responses by increasing B cell proliferation and Ab secretion (23). Interestingly, IFN-α also negatively correlated with CD95 expression on plasmablasts, which could explain why B cells treated with type I IFNs have increased survival and resistance to Fas-mediated apoptosis (46). Therefore, IFN-α may protect antigen-specific B cells from apoptosis by modulating CD95 expression in addition to promoting B cell proliferation. These findings are in accordance with several highly successful vaccines, such as the yellow fever vaccine, which induces a robust type I IFN response and remarkably long-lived responses (47). However, the timing and persistence of type I IFN signaling is likely critical for the beneficial effect, as prolonged signaling has been associated with exhaustion and suppression of cellular responses during malaria infection (48). Together, we have performed an extensive characterization of the responses elicited to Pfs25 in the NHP model, emphasizing the role of B cell targeting via antigen delivery, strong IFN-α polarizing adjuvants, and CD4 help for improving Ab magnitude and durability.

## Methods

For additional details, see the Supplemental Methods.

### Study design.

The aim of this study was to analyze various compartments of the immune response after immunization with formulations of the Pfs25 protein and distinct TLR-targeting adjuvants to improve immunogenicity and guide future clinical trials. Twenty-four Indian rhesus macaques (3 years of age) were sex- and weight-matched to be divided into 4 separate immunization groups with 6 animals per group. The animals were followed over time, and samples from all NHPs were used throughout the study, unless otherwise noted. No randomization or blinding was performed.

### Immunizations.

Rhesus macaques were allocated to 4 groups (*n* = 6/group), as shown in Figure 1. Animals in all groups received 50 μg Pfs25 protein on a monomer basis. Group 1–3 received SVP[Pfs25] with 500μg CpG (Gr 1), 150 μg R848 (Gr 2), or GLA-LSQ containing 12.5 μg GLA and 25 μg QS21. Immunizations were delivered in a volume of 1 ml s.c. Animals in group 4 were immunized i.m. with Pfs25-EPA containing 50 μg Pfs25 mixed with GLA-LSQ containing 25 μg GLA and 50 μg QS21 in a volume of 0.5 ml and in accordance with past protocols (16). Complete blood counts and liver function tests were performed 24 hours after the immunization (Adlego).

### Statistics.

Statistical calculations were performed in GraphPad Prism 6. Data are shown as mean ± SEM, unless otherwise noted. Comparisons between groups were determined using 2-way ANOVA with Tukey’s multiple comparison test. Differential expression of genes was calculated using a paired 2-tailed Student’s *t* test. Correlation analysis was performed using a nonparametric Spearman’s test with 2-tailed *P* value. A *P* value of less than 0.05 was considered significant.

### Study approval.

This animal study was approved by the Stockholm Ethical Committee on Animal Experiments, organized under the Swedish Board of Agriculture (permit N2/15. Indian rhesus macaques were housed in the Astrid Fagraeus laboratory at the Karolinska Institutet according to guidelines of the Association for Assessment and Accreditation of Laboratory Animal Care, and all procedures were performed according to the provisions and general guidelines of the Swedish Animal Welfare Agency.

## Author contributions

LJ, CPO, PED, EAT, UWR, AW, and KL designed research. EAT, SO, KM, KR, MS, CPO, and KL performed experiments. EAT, SO, KM, MJ, CAL, CPO, and KL analyzed data. DLN, PED, and RFH designed research and contributed adjuvants and antigens. EAT and KL wrote the paper.

## Supplementary Material

Supplemental data

Supplemental Table 1

## Version 1 05/17/2018

Electronic publication
