# Supplementary material for: TLR-adjuvanted nanoparticle vaccines differentially influence the quality and longevity of responses to malaria antigen Pfs25
Source: JCI Insight. 2018 May 17;3(10):e120692. doi: 10.1172/jci.insight.120692 (PMC6012510; doi:10.1172/jci.insight.120692)
Supplement: Supplemental data [file jciinsight-3-120692-s111.pdf]

Supplementary Materials and Methods

Vaccine Formulations

SVP were prepared according to the literature (1) . Synthetic polymers (PLGA, PLA, PLA-PEG), were purchased from Evonik Inudstries (Essen, Germany). Poly(lactic-*co*-glycolic acid)-Resiquimod (PLGA-R848), was prepared by Johnson Matthey (London, UK). CpG ODN phosphodiester 2395 (CpG), was purchased from Nitto Denko Avecia (Milford MA, USA). SVP[Pfs25] were prepared by first concentrating the recombinant Pfs25 protein (2) to approximately 12mg/ml in aqueous pH 7.5 solution using a Vivaspin 20 3k MWCO centrifuge filter device (GE Healthcare, Chicago IL, USA). The SVP[Pfs25] were then prepared using the double emulsion method(1, 12). Pfs25 was extracted from SVP[Pfs25] and quantified via reverse-phase HPLC (RP-HPLC). The SVP[Pfs25] nanoparticle concentration in solution was determined by a gravimetric method. Pfs25 load was then calculated by RP-HPLC result (recovered Pfs25), divided by the dry weight of SVP[Pfs25] in solution. The similar method was used for the CpG load, with an appropriate RP-HPLC analytical method (gradient, column, and solvents). For R848 load, the SVP[R848] was hydrolyzed under basic conditions first, then quantified using an appropriate RP-HPLC method (gradient, column, and solvents). SVPs were evaluated using drug load (as weight% of the dry nanoparticles), endotoxin via USP 38<85>, and size by polydisperity index using dynamic light scattering (DLS). Summary of SVP properties can be found in Table S2. Pfs25-EPA was manufactured at the Laboratory of Malaria Immunology and Vaccinology, NIAID/NIH, using thioether crosslinking chemistry as described previously (3) . GLA-LSQ was provided by Infectious Disease Research Institute, Seattle, WA.

Cryogenic Transmission Electron Microscopy

Cryogenic transmission electron microscopy images were prepared as described previously (4, 5) . Nanoparticles were prepared at 5 mg/mL in 0.1X PBS pH 7.4. Vitrified specimens for cryo-TEM imaging were prepared in an automated plunge freezing instrument (Vitrobot Mark IV , Thermo Fisher, Eindhoven, The Netherlands) suspending the grid (EMS, LC300-CU, Lacey Carbon film) above a

liquid nitrogen cooled dewar containing approximately 3 mL of liquid ethane. Using a laboratory adjustable pipette, 5 µL of the nanoparticle solution was applied to the grid. A paper filter (595 Filter paper, Ø 55/20 mm, Thermo Fisher, Eindhoven, The Netherlands) was applied for 2 seconds to the sample followed by immediate release of the grid into the liquid ethane to form the thin vitrified film. Observation was made at -170°C in a Tecnai F 20 microscope (Thermo Fischer, Eindhoven, The Netherlands) operating at 200 kV equipped with a cryo-specimen holder Gatan 626 (Warrendale, PA, USA).

Digital images were recorded at low dose (2000-3000 e-/nm2) with a Falcon III camera (Thermo Fisher, Eindhoven, The Netherlands), at 4098 X 4098 pixels. Magnification of 29000X (px=0.35 nm), using a defocus range of -2 µm to -3 µm. The images confirmed the spherical morphology indicated by the DLS results. SVP[R848] (prepared using the single emulsion method), are spherical, solid core nanoparticles. SVP[CpG] and SVP[Pfs25] (prepared using the double emulsion method), appear as polymersome structures with a dense thin polymer shell surrounding an aqueous core containing the Pfs25 antigen or CpG adjuvant, respectively.

Blood sample processing

Blood PBMCs were isolated using a density-gradient with Ficoll-Paque (GE Healthcare) according to standard procedures as previously described (6) . PBMCs were washed and maintained in complete media (R10; RPMI 1640/10% FCS/100 U penicillin/0.1 mg streptomycin; HyClone), or frozen in 90% heat-inactivated FBS and 10% DMSO (Sigma-Aldrich) and stored at -150C. Experiments were performed on both fresh and frozen cells.

Phenotypic analysis

For innate studies, 24 hours following immunization, $5x10^6$ cells were stained with LIVE/DEAD Fixable Blue Dead Cell kit according to manufacturers protocol (Invitrogen) and blocked with FcR-blocking reagent (Miltenyi). Samples were then surfaced stained with a panel of fluorescently labeled Abs (Supplemental Table 3) to

determine cell proportions and maturation. Where indicated, numbers of specific cell subsets are normalized to complete blood count (CBC) by multiplying the subset's percent of monocyte or lymphocyte gate by CBC number of monocytes or lymphocytes/ml respectively. For plasmablast phenotyping, $2x10^6$ cells were stained with LIVE/DEAD Fixable Blue Dead Cell kit and surfaced stained with a panel of fluorescently labeled Abs (Supplemental Table 3). Cells were then fixed and permeabilized using Cytofix/Cytoperm solution (BD) followed by intracellular staining with IgG FITC (BD, clone G18-145) and Ki67 PE (BD, clone B56).

Cytokine/chemokine secretion analysis

Rhesus plasma samples were evaluated for systemic cytokine and chemokine secretion using the 30plex ProcartaPlex NHP Cytokine & Chemokine Panel (ebioscience) and CXCL13 ELISA (R&D Systems). All assays were performed according to manufacturer's protocol.

RNA extraction

Blood was collected in Paxgene tubes (PreAnalytiX) and stored at –20°C until use. The tubes were thawed at room temperature over night and then centrifuged for 10 minutes at 3000 x g using a swing-out rotor. Supernatant was removed. 4 ml H_2O was added to the pellet, the tube was then vortexed until the pellet was visibly dissolved. Tubes were centrifuged for 10 minutes at 3000 x g using a swing-out rotor, supernatant was discarded. 350 µl resuspension buffer (BR1) was added followed by vortex until the pellet was visibly dissolved. Samples were then loaded into the QIAcube shaker and the protocol "PAXgene Blood RNA Part A" was run followed by the protocol "PAXgene Blood RNA Part B". (PAXgene® Blood RNA Kit Handbook, PreAnalytiX, Qiagen). The concentration (OD260) and purity (OD260/280 ratio) of extracted total RNA was measured using NanoDrop ND-1000 spectrophotometer (Thermo Fisher Scientific). Agilent 2200 Tapestation **(**Agilent Technologies) was used to assess the RNA integrity using the RNA ScreenTape assay according to the manufacturer's instructions. Two samples (From group 1 and

group 4) did not have high quality RNA after storage and therefore were excluded from all microarray analysis.

Microarray and data analysis

Cyanine-3 (Cy3) labeled cRNA was prepared from 200ng total RNA with Quick Amp Labeling Kit (Agilent Technologies) according to manufacturer's protocol, followed by RNeasy column purification (Qiagen). 1.65 μg Cy3-cRNA was fragmented at 60°C for 30 min in fragmentation buffer and blocking agent (Agilent Technologies) as per manufacturer's instructions. Agilent hybridization buffer was added to the fragmentation mixture and hybridized to Agilent Rhesus Macaque Gene Expression Microarrays v2 (part number G2519F-026806) for 17h at 65°C in a rotating Agilent hybridization oven. Microarrays were first washed with GE Wash Buffer1 and then with +37°C GE Wash buffer 2 (Agilent Technologies). Slides were immediately scanned using Agilent DNA microarray scanner G2505C at 3μm resolution (Agilent Technologies). The fluorescent intensities of the scanned images were extracted and preprocessed with the Agilent Feature Extraction Software (version 10.7.3.1). Local background adjusted signals (gProcessedSignal) were further quantile normalized using the R/Bioconductor package to achieve consistency between samples. Control probes were removed prior to quantile normalization. Data was filtered to only include genes that were detected in all samples of at least one group and probes without associated GeneID were excluded. Microarray data has been submitted to National Center for Biotechnology Information GEO (accession no. GSE102909).

Normalized data was analyzed to identify statistically significant genes using a paired two-tailed Students t-test. Probes were selected for further analysis if FC > 2 and a raw p-value < 0.01. For functional pathway analysis, all genes were ranked by paired average FC with respect to baseline for each vaccination group. Pre-ranked GSEA was performed with 1000 permutations using the Java interface (Broad Institute, software.broadinstitute.org/gsea/index/jsp) with BTMs as the gene set (7) and BTMs with less than 10 genes were excluded. Modules enriched with a p-value <0.05 were considered significant.

Pfs25 Ab ELISA and avidity assay

Basic methodology of regular ELISA has been described elsewhere (8) . All ELISA plates were coated with unconjugated Pfs25 protein that was produced in the *Pichia pastoris* expression system (9) . ELISA reference standard was generated by pooling anti-Pfs25 antisera from all monkeys collected at Week 6, and a relative antibody level (i.e., ELISA units) of test sample was determined based on a standard curve generated with a serially dilution of the ELISA reference standard.

To determine the avidity of anti-Pfs25 antibodies, antisera (i.e., primary antibodies) were diluted to 2 ELISA units. After primary antibody incubation, an additional incubation step with varying concentrations of urea (from 0 to 8 M) in Tris-buffered saline for 15 minutes was performed. The remainder procedure was the same as regular ELISA. Since many antisera showed strong avidity, the concentration of urea resulting in 80% of the original OD value (IC_{20}) was calculated using linear regression. All of the data sets fit the linear regression models well ($r^2>0.85$, data not shown).

Antibody half-life

Nonlinear mixed models were used for assessment of antibody titer half-life separately in two decay periods: between weeks 6-16 following the first boost, and between weeks 18-46 following the second boost. Conditional on random effects for β_0 and β_1, we fit an exponential decay model:

$$y = (\beta_1 - \beta_0)e^{-\beta_2 t} + \beta_0 + \varepsilon$$

to titer data at- and post- peak separately for each study group and decay period, where y is the observed antibody titer, t is the number of weeks elapsed since the peak titer measurement, β_1 is the mean peak titer at time t = 0, β_0 is the mean titer set-point, β_2 is the mean exponential rate of decay, and ε is a normally distributed error. Fixed effects were estimated using the maximum likelihood method implemented in *R*'s nlme function.

An animal's titer half-life was estimated as:

$$\log\{(\hat{\beta}_1-\hat{\beta}_0)/(\hat{\beta}_1/2-\hat{\beta}_0)/\hat{\beta}_2\ weeks\}$$

During the decay period from weeks 6-16 one animal's antibody half-life (Group 1) was non-estimable because the fitted model's set-point titer was greater than its peak, therefore this animal was excluded from analysis during this time point.

B cell ELISpot

The frequency of Pfs25-specific Ab secreting cells (ASCs) and memory B cells was determined as previously described (10) , with some modifications. In brief, MAIPSWU10 96-well plates (Millipore) were coated with 10 µg/ml of anti-human IgG (Fcγ; Jackson ImmunoResearch Laboratories), Pfs25 protein, or ovalbumin (Invivogen). PBMCs were transferred in duplicate dilution series and cultured overnight at 37°C. For enumeration of ASCs, PBMCs were plated directly without prior stimulation, whereas for memory B cells PBMCs were pre-stimulated for 4 days at $2x10^6$ cells/ml with 5 µg/ml CpG-B (ODN 2006; Invivogen), 10 µg/ml Pokeweed mitogen (PWM; Sigma-Aldrich), and 1:10000 Protein A from *Staphylococcus aureus* Cowan strain (SAC; Sigma- Aldrich). Plates were washed with PBS containing 0.05% Tween-20 (PBS-T), then incubated with 0.25 µg/ml biotinylated goat anti-human IgG (Fcγ; Jackson ImmunoResearch Laboratories) in PBS-T, washed and incubated with streptavidin-conjugated alkaline phosphatase (Mabtech) diluted 1:1000 in PBS-T. Spots were developed with BCIP/NBT substrate (Mabtech) and counted using an AID ELISpot Reader and version 6 of the accompanying software (Autoimmun Diagnostika). Unspecific spots were subtracted from antigen-specific wells, as defined by spots counted in samples incubated in ovalbumin-coated wells.

SMFA

The SFMA was performed using Protein G-purified total IgGs as described previously (11) . In brief, 0.15%± 0.05% mature Stage V gametocytes (*P. falciparum* strain NF54) were mixed with 7.5 mg/ml of purified IgG, then fed to 4-6 day old

female *A. stephensi*. After 7 days, midguts from mosquitoes with eggs at the time of dissection (n=20 per group) were evaluated and oocysts number in each midgut was recorded. Percent reduction in infection intensity was calculated relative to the pooled Week 0 IgGs from all monkeys tested in the same assay.

Antigen recall T cell assay

For assessment of Pfs25-specific T cells, $1.5x10^6$ PBMCs were cultured in 200ul of R10 per stimulation in a 96 well plate. The cells were stimulated with overlapping peptides (15mers overlapping by 11 amino acids) spanning the entire Pfs25 protein in the presence of 10μg/ml brefeldin A (Life Technologies) overnight. Samples were stained the following morning to evaluate cytokine production and phenotype. Cells were first stained with LIVE/DEAD Fixable AquaBlue viability dye (Invitrogen), surface stained, fixed and permeabilized (BD, Cytofix/Cytoperm), and then intracellularly stained (Supplemental Table I). The specificity of the OL peptides was confirmed side by side with whole Pfs25 protein. As expected there was a strong correlation for T cell responses whether stimulated with whole protein or peptide. Background cytokine staining was subtracted, as defined by staining in the matched DMSO only controls.

References

1. Astete C, Sabliov C (2006) Synthesis and characterization of PLGA nanoparticles. *Journal of Biomaterials Science, Polymer Edition* 17:247–289.
2. Tsai CW, Duggan PF, Shimp RL, Miller LH, Narum DL (2006) Overproduction of Pichia pastoris or Plasmodium falciparum protein disulfide isomerase affects expression, folding and O-linked glycosylation of a malaria vaccine candidate expressed in P. pastoris. *J Biotechnol* 121:458–70.
3. Shimp RL et al. (2013) Development of a Pfs25-EPA malaria transmission blocking vaccine as a chemically conjugated nanoparticle. *Vaccine* 31:2954–62.
4. Hajibagheri M, Harris J, Adrian M (1999) *Electron Microscopy Methods and Protocols*.
5. Morabito KM et al. (2016) Intranasal administration of RSV antigen-expressing MCMV elicits robust tissue-resident effector and effector memory CD8+ T cells in the lung. *Mucosal Immunol*.
6. Thompson EA et al. (2015) Human Anti-CD40 Antibody and Poly IC:LC Adjuvant Combination Induces Potent T Cell Responses in the Lung of Nonhuman

Primates. *J Immunol* 195:1015–24.
7. Li S et al. (2014) Molecular signatures of antibody responses derived from a systems biology study of five human vaccines. *Nat Immunol* 15:195–204.
8. Miura K et al. (2008) Development and characterization of a standardized ELISA including a reference serum on each plate to detect antibodies induced by experimental malaria vaccines. *Vaccine* 26:193–200.
9. Zou L, Miles AP, Wang J, Stowers AW (2003) Expression of malaria transmission-blocking vaccine antigen Pfs25 in Pichia pastoris for use in human clinical trials. *Vaccine* 21:1650–7.
10. Sundling C et al. (2010) Soluble HIV-1 Env trimers in adjuvant elicit potent and diverse functional B cell responses in primates. *J Exp Med* 207:2003–17.
11. Miura K et al. (2013) Qualification of standard membrane-feeding assay with Plasmodium falciparum malaria and potential improvements for future assays. *PLoS ONE* 8:e57909.

Supplemental Figure 1

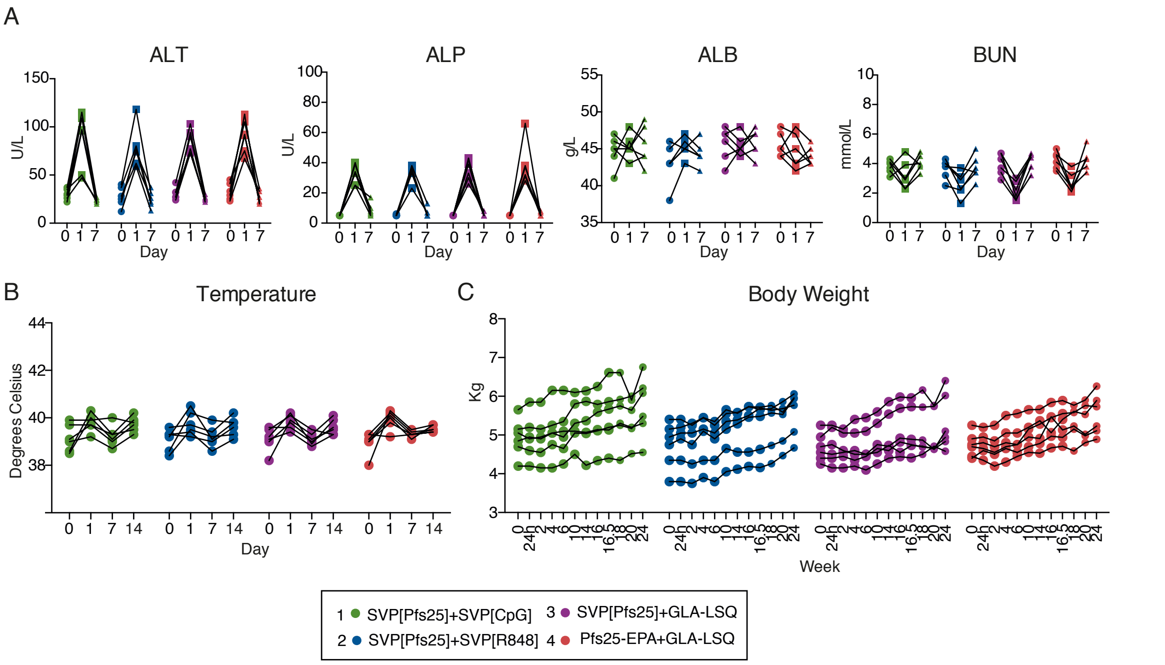

Figure S1: All vaccine formulations were well tolerated
(A) Liver function tests: Alanine amino transferase (ALT), Alkaline phosphatase (ALP), Albumin (ALB), Blood urea nitrogen (BUN), taken at day 0, 1, and 7 post prime immunization. (B) Body temperature (degrees celsius) taken at day 0, 1, 7, and 14 post prime immunization. (C) Body weight (kg) measured at blood draw throughout study.

Supplemental Figure 2

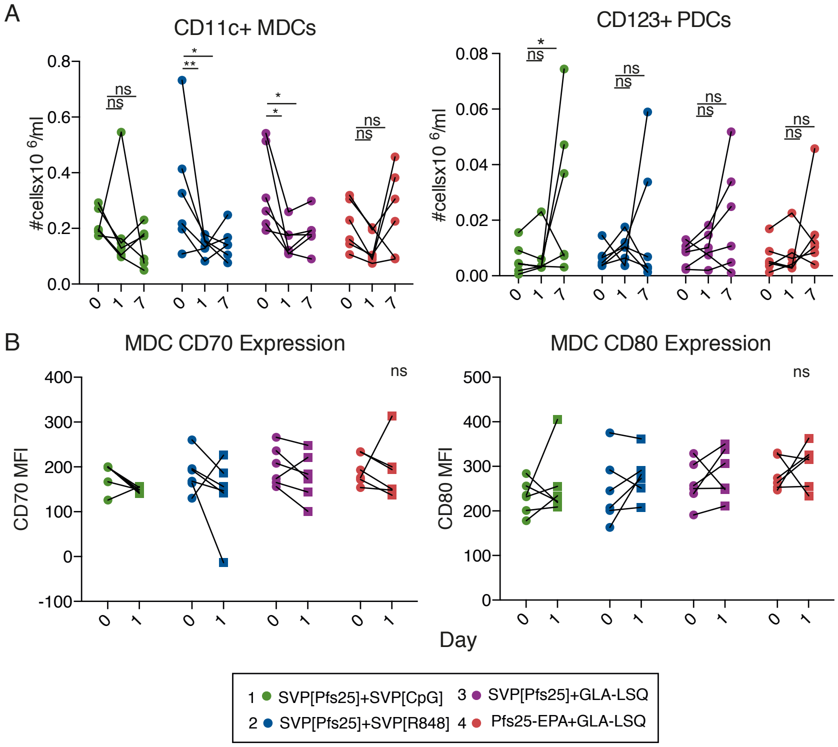

Figure S2: Immunization induces limited activation of DC subsets
(A) Cell subset frequencies normalized to monocyte complete blood counts. (B) CD11c+ MDC activation as assesed by CD70 and CD80 expression (MFI values shown).

Supplemental Figure 3

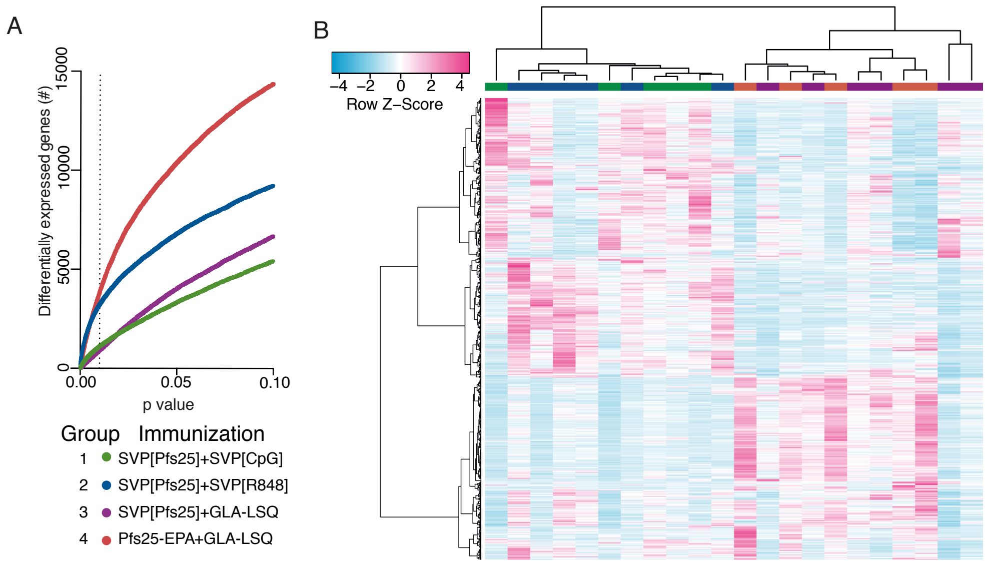

Figure S3: Adjuvant driven modulation of gene expression
(A) Number of differentially expressed genes 24 hours after immunization compared to baseline using different paired t-test two-tailed p-values, dotted line represents p-value cut off of 0.01. (B) Heatmap depicting average fold change (FC) values of selected genes (p-value<0.01, FC>2) for each individual animal with two-way hierarchical clustering.

Supplemental Figure 4

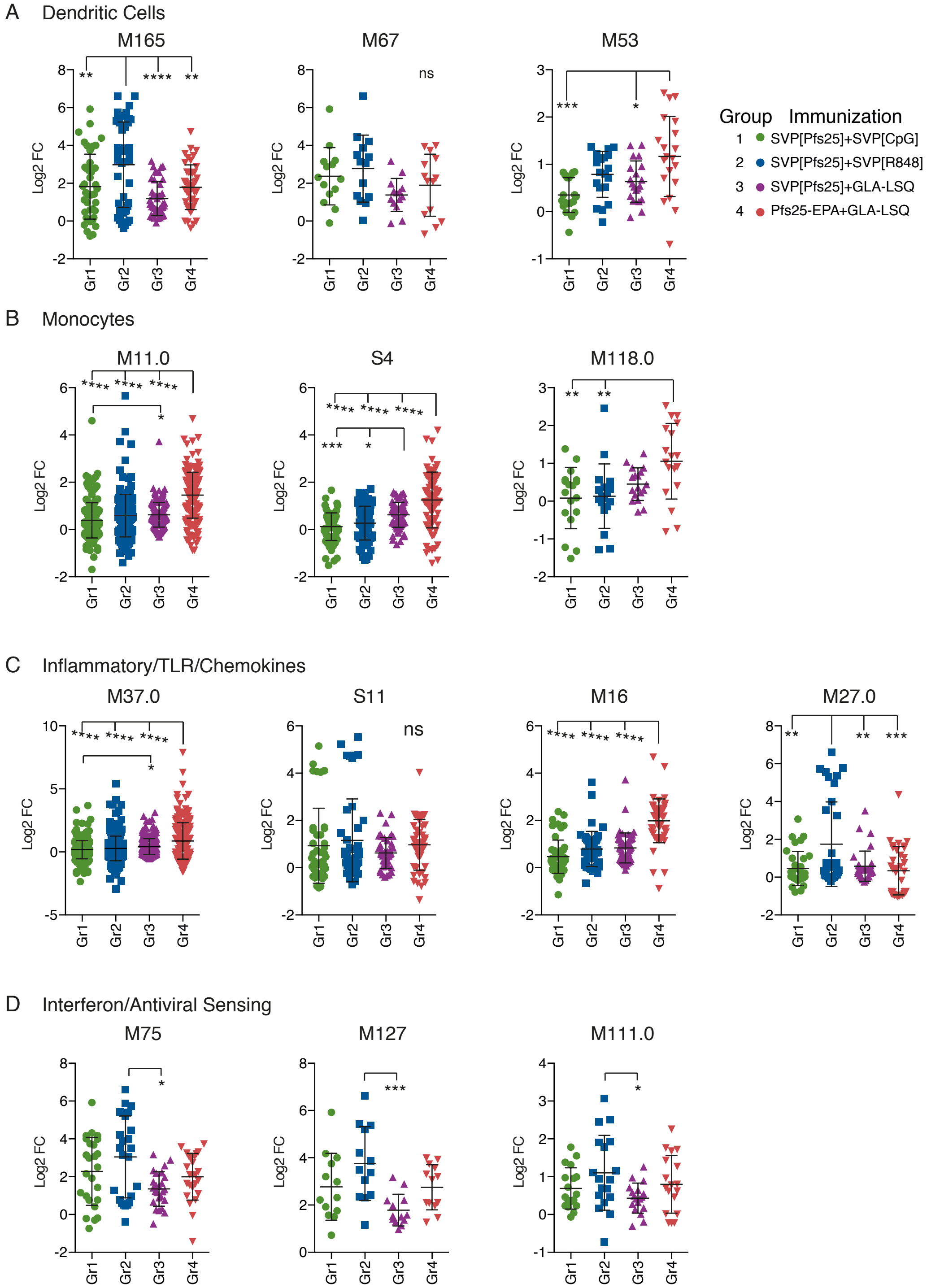

Figure S4: Average fold change values of blood transcript modules
Average fold change (FC) value per group from day 0 to day 1 for each gene contained within blood transcript modules with higher level annotation of Dendritic Cells (A), Monocytes (B), Inflammatory/TLR/Chemokines (C), or Interferon/Antiviral Sensing (D). All data represented as mean±SEM unless otherwise noted. Groups were compared using two-way ANOVA. $^*p \leq 0.05$; $^{**}p \leq 0.01$; $^{***}p \leq 0.001$; $^{****}p \leq 0.0001$

Supplemental Figure 5

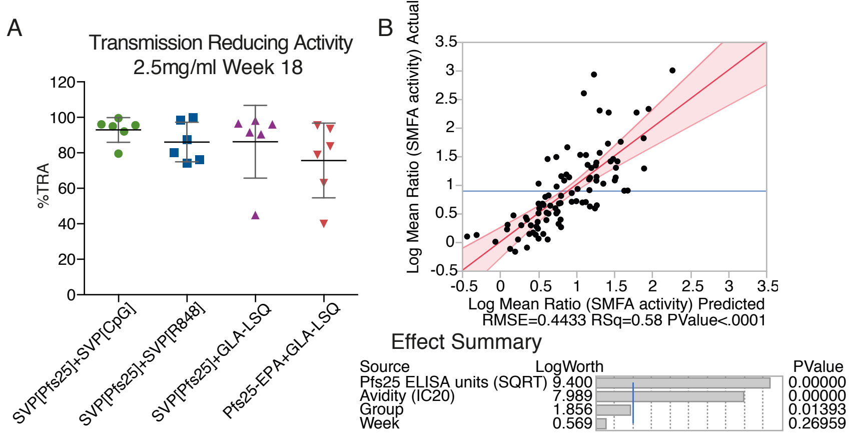

Figure S5: SMFA activity

(A) Standard Membrane Feeding Assay (SMFA) performed using 2.5mg/ml purified IgG from week 18 samples (mean±SEM). (B) Multiple linear regressison model to describe SMFA activity (Log Mean Ratio).

Supplemental Table 2: SVP formulation summary

Formulation	Antigen/Adjuvant	Load (wt%)	Endotoxin (EU/mL)	Size (nm)	PDI (RU)
SVP[CpG]	CpG (PO 2395)	4.85	<5	129	0.078
SVP[R848]	Polymer-R848	4.51	<5	147	0.046
SVP[Pfs25]	Pfs25	0.82	<5	139	0.019

Supplemental Table 3: Fluorescently labeled antibodies for flow cytometry

Innate Phenotyping			Plasmablast Phenotype			T Cell Stimulation		
**Marker**	**Clone**	**Supplier**	**Marker**	**Clone**	**Supplier**	**Marker**	**Clone**	**Supplier**
CD123	7G3	BD Biosciences	CD95	DX2	BD Biosciences	CD45RA	5H9	BD Biosciences
CD40	5C3	Biolegend	CD20	2H7	Biolegend	CD4	S3.5	Invitrogen
CD11c	3.9	BioLegend	CD3	SP34-2	BD Biosciences	ICOS	C398.4A	BioLegend
CD70	TU36	BD Biosciences	CD11c	M5E2	Biolegend	CD8	RPA-T8	BioLegend
NKg2a	Ki-24	Beckman Coulter	CXCR3	3.9	Biolegend	CCR7	GO43H7	BioLegend
CD3	IM3291U	BD Biosciences	CD14	6H6	Biolegend	CXCR3	1C6/CXCR3	BD Biosciences
CD66	SP34-2	Miltenyi	CD123	Tü36	Biolegend	PD-1	EH12.2H7	BioLegend
CD20	TET2	BioLegend	HLA-DR	2D10	Life Tech	IL-5	JES1-39D10	BD Biosciences
CD16	M5E12	BioLegend	CD80	12G5	Biolegend	IL-4	MP4-25D2	BD Biosciences
CCR7	2H7	Biolegend	CXCR4	G025H7	Biolegend	CXCR5	MU5UBEE	eBioscience
HLA-DR	2D10	Invitrogen	IgG	G180145	BD Biosciences	CD69	TP1.55.3	Beckman Coulter
CD14	38G	Biolegend	Ki67	B56	BD Biosciences	IL-21	3A3-N2.1	BD Biosciences
CD80	G043H7	BD Biosciences				IFN-g	B27	BioLegend
						CD3	SP34.2	BD Biosciences
						IL-13	JES10-5A2	BD Biosciences
						IL-17A	BL168	BioLegend
						IL-2	MQ1-17H12	BD Biosciences
